# Zusanli (ST36) Acupoint Injection With Dexamethasone for Chemotherapy-Induced Myelosuppression: A Systematic Review and Meta-Analysis

**DOI:** 10.3389/fonc.2021.684129

**Published:** 2021-07-06

**Authors:** Jiangfeng Chen, Zhixian Lin, Jiyuan Ding

**Affiliations:** ^1^ Department of Integrated Traditional Chinese & Western Medicine Oncology Ward 1, Hangzhou Cancer Hospital, Hangzhou, China; ^2^ Department of Oncology, The Third People’s Hospital of Hangzhou, Hangzhou, China

**Keywords:** chemotherapy, myelosuppression, zusanli (ST36), acupoint injection, dexamethasone, meta-analysis

## Abstract

**Objective:**

Myelosuppression is the most common adverse reaction of chemotherapy, which seriously affects the course of treatment. Zusanli (ST36) acupoint injection with dexamethasone has achieved good clinical efficacy in China. This study aimed to systematically evaluate the efficacy of ST36 acupoint injection with dexamethasone in the treatment of chemotherapy-induced myelosuppression (CIM).

**Methods:**

Randomized controlled trials of CIM treated with ST36 acupoint injection with dexamethasone were retrieved from eight electronic databases. We used the Cochrane Collaboration tool to assess the risk of bias. Excel 2010 was used to establish a database for information extraction, and RevMan 5.3.0 software was used to analyze the included test data. GRADE profiler 3.6 software was used to grade the quality of evidence for the outcome indicators of the study.

**Results:**

A total of 17 studies involving 1177 patients were included in this meta-analysis. The results showed that, compared with conventional western medicine (CWM), ST36 acupoint injection with dexamethasone could significantly improve the clinical total effective rate [RR=1.95, 95% CI (1.53, 2.49), P <0.00001] and increase white blood cell (WBC) (MD=1.38, 95% CI (0.74, 2.01), P<0.0001) and hemoglobin (Hb) levels [MD=3.89, 95% CI (1.57, 6.20), P=0.001]. In addition, ST36 acupoint injection with dexamethasone can shorten recovery time of myelosuppression [MD=-3.94, 95% CI (-4.97 to -2.91), P<0.00001] and improve Karnofsky performance status [MD=10.7, 95% CI (1.36, 20.05), P=0.02<0.05]. However, there was no significant difference among ST36 acupoint injection with dexamethasone and CWM in platelet (PLT) elevation [MD=4.61, 95% CI (-10.14, 19.35), P=0.54].

**Conclusion:**

This study found that ST36 acupoint injection with dexamethasone had a positive effect on CIM. However, more studies with well-designed, large sample size, strict randomization, and clear descriptions about detection and reporting processes are needed in the future to further confirm the efficacy of ST36 acupoint injection with dexamethasone in the treatment of CIM.

**Systematic Review Registration:**

https://www.crd.york.ac.uk/, identifier CRD42021223979.

## Introduction

Chemotherapy is one of the main treatments for malignant tumors. Most chemotherapy drugs destroy normal bone marrow cells while killing tumor cells, leading to different degrees of myelosuppression, resulting in a decrease in white blood cells (WBC), hemoglobin (Hb), and platelet (PLT), and increasing the risk of infection, anemia, or hemorrhage in patients with cancer, and in severe cases, can be life-threatening (1, 2). These conditions often lead to treatment delay, dose reduction, or discontinuation, which may ultimately affect long-term clinical outcomes and reduce the disease-free survival rate and overall survival rate (3–5). Recombinant human granulocyte colony stimulating factor (rhG-CSF), recombinant human thrombopoietin (rHuTPO), recombinant human erythropoietin (rhEpo), PLT, and red blood cell (RBC) transfusion, and so on are often used clinically to improve Chemotherapy-induced myelosuppression (CIM). Although the above drugs take effect quickly, they are expensive and have many adverse reactions. For example, granulocyte macrophage colony-stimulating factor can cause shock or chronic fibrous pneumonia, and repeated PLT transfusions can lead to the formation of alloantibodies (6). Therefore, it is very important to find a safe, effective, and inexpensive treatment to relieve CIM.

Acupoint injection is a technique of acupoint stimulation, in which drugs are injected into certain acupoints to achieve a longer-lasting effect than traditional acupuncture or simple intramuscular injection (7, 8). Also, it is also used in the treatment of CIM in China (9). According to traditional Chinese medicine, CIM belongs to “deficiency of fatigue” disease, and the syndrome is a type of deficiency of both qi and blood. Zusanli is a commonly used health care point in Chinese medicine, and it is located 3 cun below the lower border of the patella, one fingers’ breadth lateral to the anterior crest of the tibia, in the tibialis anterior muscle (10). “Cun” is defined in acupuncture as the width of the patient’s thumb interphalangeal joint (11). Zusanli has the effects of regulating spleen and stomach, replenishing qi and nourishing blood, and improving weakness (12, 13). Its traditional therapeutic properties are very suitable for the treatment of CIM. Moreover, modern studies have found that the branches of microvascular, nerve, and lymphatic vessel in the Zusanli acupoint area were abundant. Stimulating Zusanli can achieve a bi-regulation effect on the human neuro-immune-endocrine network (14), which has a benign regulation effect on myelosuppression and can improve the hematopoietic function of the bone marrow system (15). In addition, the glucocorticoid dexamethasone can stimulate the secretion of vasoactive substances, thereby stimulating the hematopoietic function of bone marrow (16). ST36 acupoint injection with dexamethasone is based on the theory of traditional Chinese medicine meridian and combined with modern pharmacology to treat CIM. It has the dual effects of acupuncture and drugs. However, there are no systematic review and meta-analysis on the ST36 acupoint injection with dexamethasone in the treatment of CIM. Therefore, we conducted the first systematic review and meta-analysis based on a randomized-controlled trials (RCTs) to evaluate whether ST36 acupoint injection with dexamethasone can treat CIM.

## Methods

### Study Registration

This systematic review and meta-analysis followed the PRISMA guidelines and were registered in the PROSPERO database (https://www.crd.york.ac.uk/). The registration number is CRD42021223979.

### Search Strategy

We searched PubMed, Web of Science, the Cochrane Library, Embase, Sinomed, China National Knowledge Infrastructure (CNKI), Chinese Scientific Journal Database (VIP), and Wanfang Database. The search time was from establishment to March 1, 2020. The following search terms were used: “Zusanli,” “ST36,” “Acupoint Injection,” “Dexamethasone,” “Chemotherapy,” “Myelosuppression,” “leukocytopenia,” “thrombocytopenia,” “decreased hemoglobin,” “anemia.” Details of the search strategies were available in Supplementary materials 1. There were no restrictions on language and publication status. In addition, we searched the conference literature and clinical registration data for more possible related trials. The search was conducted independently by two authors (JC and ZL).

### Inclusion and Exclusion Criteria

The inclusion criteria were as follows: 1) Study design: All included studies were RCTs. 2) Participants: Patients diagnosed as myelosuppression after chemotherapy according to WHO grading criteria for toxic reactions to chemotherapy (17) were not restricted by age, gender, race, tumor type, chemotherapy regimen, and dose of chemotherapy drugs. 3) Interventions: Patients in the trial group received ST36 acupoint injection with dexamethasone combined with or without conventional western medicine (CWM). The specific operation method was to pierce the injection needle into the Zusanli acupoint and then slowly inject the dexamethasone solution after the patient feels “deqi.” “Deqi” was a traditional acupuncture term used to describe the meridian-qi induction generated at the acupuncture site, and patients generally experience sensations, such as acid, numbness, and heaviness (18, 19). There were no restrictions on drug dosage or duration of treatment. The control group was treated with CWM, such as rhG-CSF, recombinant human interleukin-11 (rhIL-11), rHuTPO, leucogen tablets, and other symptomatic treatments. There were no restrictions on the dosage or dosage form (oral or injectable drugs) or duration of treatment. 4) Outcomes: the primary outcome was effective rate, which was defined as the WBC count≥4.0×10^9/L and/or PLT count≥75×10^9^/L. Secondary outcomes were comparison of blood cell count after treatment, blood cell recovery time, and quality of life (Karnofsky Performance Status, KPS) score. The included studies reported at least one of the abovementioned outcomes.

The exclusion criteria were as follows (1): full text cannot be obtained through electronic search, manual search, and author’s mailbox (2); repeated publication (3); non-randomized controlled trials such as meta-analysis, retrospective studies, case reports, experimental studies, and conference abstracts research (4); patients with myelosuppression after receiving radiotherapy or combined chemotherapy (5); acupoint injection drugs in the trial group were dexamethasone solution combined with other solutions or non-dexamethasone solutions (6); The acupoints injected in the trial group were Zusanli combined with other acupoints or non-Zusanli acupoints (7); Experiments that lack result data or cannot be analyzed.

### Study Selection and Data Extraction

Study selection and data extraction were performed independently by two researchers (JC and ZL), and all differences were resolved by a third researcher (JD). We have extracted the following information from the included studies: general information (first author, year of publication), characteristics of participants (age, gender, sample size, types of cancer), intervention measures and comparative details (treatment time, dosage of medication, etc.), and outcomes. If sufficient data cannot be extracted from eligible studies, we will contact the corresponding author.

### Risk of Bias Assessment

Two researchers (JC and ZL) used the Cochrane Collaboration’s risk of bias tool (20) to independently assess the risk of bias of included studies. The assessment contents of the bias risk assessment tool include the following: random sequence generation (selection bias), allocation concealment (selection bias), blinding of participants and personnel (performance bias), blinding of outcome assessment (detection bias), incomplete outcome data (attrition bias), selective reporting (reporting bias), and other bias. After assessing the risk of bias, each item can be classified as “high risk of bias,” “low risk of bias,” and “risk of unclear bias.” If there was a difference in the two researchers’ judgment, it can be resolved through discussion with a third researcher.

### Data Analysis

The RevMan (Review Manager 5.3) statistical software provided by the Cochrane Collaboration was used for systematic evaluation and meta-analysis (21). Continuous data were expressed as mean difference (MD) with 95% confidence interval (CI). Dichotomous data were expressed as risk ratio (RR) with 95% CI. Chi-square test and I-square (I^2^) index were used to test the heterogeneity. When P≥0.05 and I^2^ ≤ 50%, it can be considered that the heterogeneity between the studies is small, and the fixed-effect model was selected. Otherwise, the random-effects model was selected. P ≤ 0.05 was considered statistically significant, and all tests were two-sided tests. In addition, for single outcome, we will draw the funnel plot to identify publication bias if the number of studies analyzed is more than 10 (22). Moreover, we will also perform the Egger’s tests through STATA v16.0 to further detect publication bias (23). Sensitivity analysis evaluated the stability of the results by removing individual studies.

### Grade Evaluation

Based on the results of the meta-analysis, the GRADE system recommendation method was used to evaluate the quality of evidence, which was divided into very low-, low-, medium-, and high-quality grades. Factors affecting the quality of evidence include the limitations of the research design and implementation process, the indirectness of the evidence, consistency of research results, accuracy of effect estimation, and publication bias. Grade Profiler 3.6 software was used for editing and analysis.

## Results

### Study Selection

**Figure 1:**
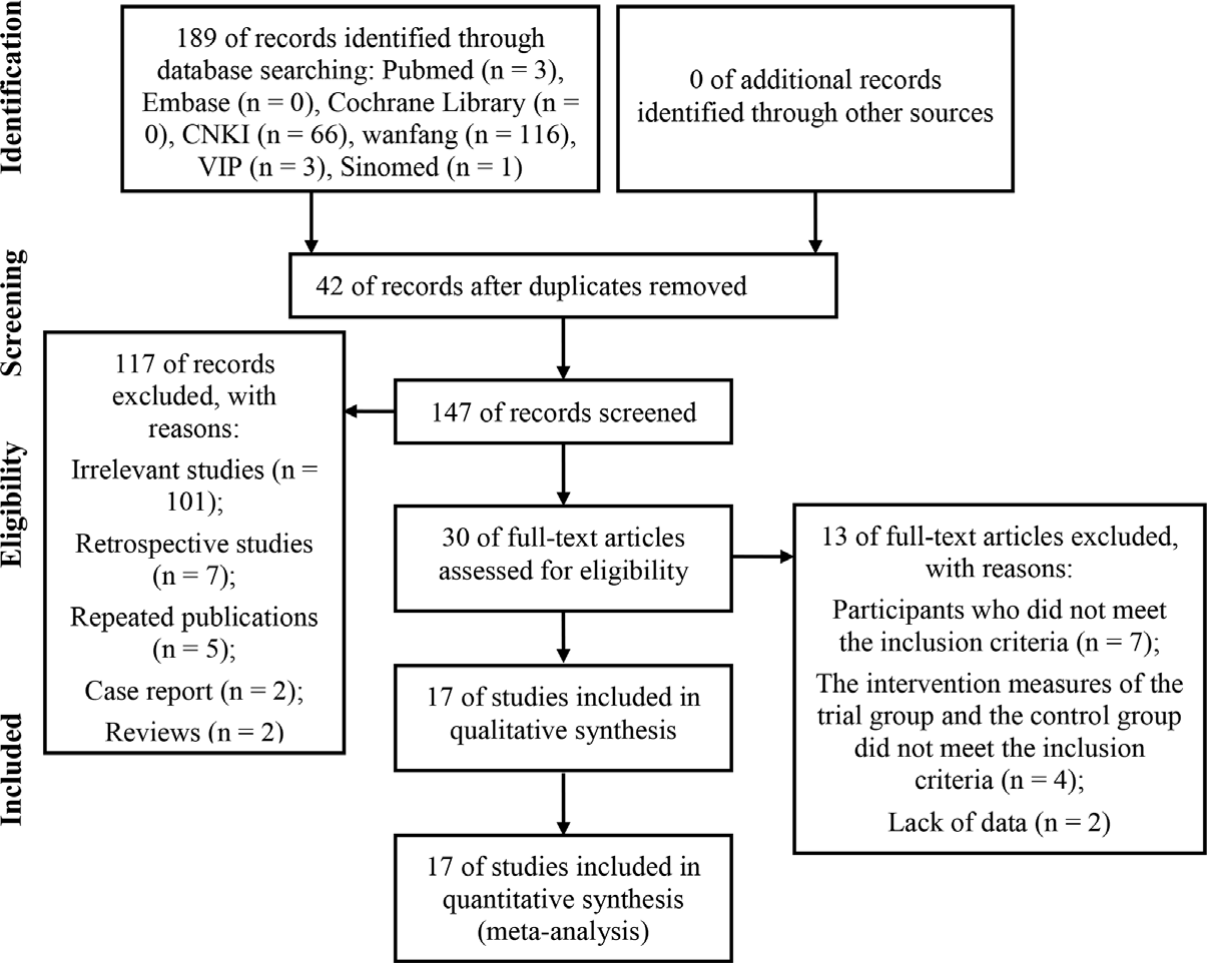
Flowchart of study identification and selection.

A total of 189 potentially relevant articles were retrieved from eight electronic databases. Among them, 42 articles were duplicated and were therefore excluded. After reviewing the titles and abstracts, 117 articles that did not meet our full-text review criteria were deleted (101 irrelevant studies, seven retrospective studies, five repeated publications, two case reports, and two reviews). The remaining 30 articles were all downloaded in full. After reading the full text, 13 studies were further deleted because of at least one of the following reasons: participants who did not meet the inclusion criteria (n = 7); the intervention measures of the trial group and the control group did not meet the inclusion criteria (n = 4); lack of data (n = 2). In the end, we included 17 eligible studies for a comprehensive analysis. The screening process was shown in **Figure 1**.

### Characteristics of Included Studies

A total of 17 randomized controlled trials were included in this study, involving 1,177 patients (595 in the trial group and 582 in the control group). All the studies were conducted in China over a period of 1993 to 2020. There were 10 studies (24–33) in the trial group were treated with ST36 acupoint injection with dexamethasone, and the control group received CWM. In the remaining seven studies (34–40) in the trial group were given ST36 acupoint injection with dexamethasone combined with CWM, while the control group was given CWM. The patients’ characteristics of included studies included age, gender, sample size, intervention measures, treatment time, outcomes, types of cancer, chemotherapy regimens, and were summarized in **Table 1**.

**Table 1:** Baseline characteristics of included studies.

First author (year)	Age/years	Gender		Sample size		Intervention measures		Treatment time(days)	Outcomes	Types of cancer	Chemotherapy regimens
		Male	Female	Trial group	Control group	Trial group(usage)	Control group(usage)				
Geng et al., 1999	20-60	72	64	68	68	DMX(acupoint Injection), bilateral, 2.5mg	Zaizhangshengxue tablets(oral) or Zhenqi Granule(oral) or Leucogen tablets(oral)	7	Effective Rate	NR	NR
Huang et al., 2012	NP	0	57	29	28	DMX(acupoint Injection), bilateral, 0.5ml+rhG—CSF(ih)	rhG—CSF(ih)	5	1.Effective Rate 2.Recovery Time	Breast cancer	chemotherapy regimens containing pirubicin, epirubicin, taxol, docetaxel
Xie et al., 2013	23-65	49	31	40	40	DMX(acupoint Injection), 5mg	rhG—CSF(ih)	5	Effective Rate	NR	NR
Yao et al., 2012	41-75	38	22	30	30	DMX(acupoint Injection), bilateral, 2mg +Leucogen tablets(oral), Berbamine hydrochloride tablets(oral)	Leucogen tablets(oral), Berbamine hydrochloride tablets(oral)	7	1.Effective Rate 2. Blood routine Levels	Lung cancer, stomach cancer, breast cancer, bowel cancer, ovarian cancer, esophageal cancer and lymphoma, etc	NR
Zhang et al., 2013	41-71	0	40	20	20	DMX(acupoint Injection)+ Diyushengbai tablet(oral) or Tabellae batiloli(oral)	Diyushengbai tablet(oral) or Tabellae batiloli(oral)	7	Effective Rate	Cervical cancer, breast cancer, ovarian cancer, pelvic malignancy	docetaxel+ cisplatin, docetaxel+ carboplatin, docetaxel+ Epirubicin, docetaxel+ nedaplatin, oxaliplatin+ etoposide, cisplatin+ etoposide
Yang et al., 2004	27-71	35	23	29	29	DMX(acupoint Injection), bilateral, 2.5mg + Chinese herb (oral)	DMX(im) + Chinese herb (oral)	7	1.Effective Rate 2.Blood routine Levels	Malignant lymphoma, lung cancer, colorectal cancer, breast cancer, ovarian cancer, nasopharyngeal cancer, cervical cancer, esophageal cancer, stomach cancer, larynx cancer, renal cancer, cancerous pleural fluid, leiomyosarcoma, multiple myeloma, liver cancer, melanoma, seminoma, osteosarcoma, prostate cancer, cervical metastatic cancer	NR
Wang et al., 2016	31-68	27	42	42	27	DMX(acupoint Injection), 5mg + rhIL-11(ih)	rhIL-11(ih)	NR	1.Effective Rate 2.Platelet levels 3.Recovery Time	NR	NR
Yu et al., 2007	23-68	39	21	30	30	DMX(acupoint Injection), bilateral, 2.5mg	Diyushengbai tablet(oral)	7	Effective Rate	Lung cancer, liver cancer, malignant lymphoma, stomach cancer, ovarian cancer, breast cancer	NR
Chen et al., 2005	21-65	39	21	30	30	DMX(acupoint Injection), bilateral, 2.5mg + Berbamine hydrochloride tablets(oral), Tabellae batiloli(oral)	rhG—CSF(ih)+Berbamine hydrochloride tablets(oral), Tabellae batiloli(oral)	5	Effective Rate	NR	NR
Lu, 2003	27-79	31	29	29	31	DMX(acupoint Injection), bilateral, 5mg + Berbamine hydrochloride tablets(oral), Leucogen tablets(oral)	Berbamine hydrochloride tablets(oral), Leucogen tablets(oral)	5	Effective Rate	NR	NR
Zhu et al., 2014	27-69	0	80	40	40	DMX(acupoint Injection), 5mg + Berbamine hydrochloride tablets(oral), Leucogen tablets(oral)	Berbamine hydrochloride tablets(oral), Leucogen tablets(oral)	7	1.Effective Rate 2.Blood routine Levels	Breast cancer	CEF
Zheng, 2010	25-76	58	54	55	57	DMX(acupoint Injection), 5mg	rhG—CSF(ih)	5	Effective Rate	Colorectal cancer, breast cancer, non-small cell lung cancer, stomach cancer, malignant lymphoma, nasopharyngeal cancer, pancreatic cancer, esophageal cancer, liver cancer, eyelid basal cell cancer, endometrial cancer	OLF、CAF、TP、GP、PF、GO、HLF、PLF、TP
Bu, 2020	NR	38	52	45	45	DMX(acupoint Injection), unilateral, 5mg	Leucogen tablets(oral) or Diyushengbai tablet(oral) or Fufang Ejiao Jiang(oral) or Amino—polypeptide tablets(oral)	NR	1.Effective Rate 2.Blood routine Levels 3.KPS	Lung cancer	chemotherapy regimens containing gemcitabine, pemetrexed, docetaxel, carboplatin, etc
Zhang, 1999	36-81	32	8	20	20	DMX(acupoint Injection), bilateral, 2.5mg	Leucogen tablets(oral), vitamin B4(oral)	14	Effective Rate	Lung cancer	NR
Peng et al., 2019	32-64	NR	NR	46	46	DMX(acupoint Injection), bilateral, 2.5mg	Normal saline (acupoint Injection)	7	1.Blood routine Levels 2.KPS	Cervical cancer	docetaxel+ carboplatin
Sun et al., 1993	4-68	44	16	30	30	DMX(acupoint Injection), bilateral, 2.5mg	Leucogen tablets(oral), Sodium Copper Chlorophyllin Tablets(oral), Tabellae batiloli(oral)	7	Effective Rate	NR	NR
Wang et al., 1995	18-76	15	8	12	11	DMX(acupoint Injection), bilateral, 2.5mg	Tabellae batiloli(oral), Leucogen tablets(oral)	6	Effective Rate	Lung cancer, breast cancer, malignant lymphoma, stomach cancer	NR

DMX, dexamethasone; ih, hyoraldermic injection; im, intramuscular injection; rhIL-11, recombinant human interleukin-11; rhG—CSF, recombinant human granulocyte colony stimulating factor; KPS, Karnofsky Performance Status; NR, Not reoralrted.

### Risk of Bias Assessment

**Figure 2:**
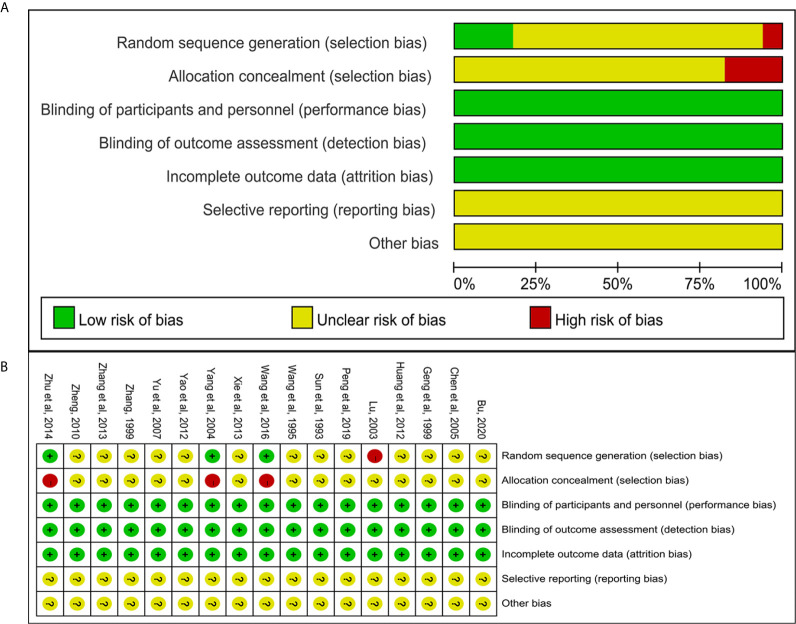
Risk of bias of included studies. **(A)** Risk of bias graph; **(B)** risk of bias summary.

We used the Cochrane risk of bias assessment tool to evaluate the quality of the studies (1). Selection bias (random sequence generation and allocation concealment), three studies (26, 37, 40) used random number table for random allocation, the risk of selection bias was considered “low”; one study (39) made distribution according to household income and payment method, the risk of selection bias was considered “high”; other studies claimed to use randomization, but no details on how to randomize were reported, the risk of selection bias was considered “unclear.” (2) Selection bias (allocation concealment): Three studies (26, 37, 40) used open random assignment table, the risk of selection bias was considered “high,” other studies had not reported allocation concealment, the risk of selection bias was considered “unclear.” (3) Performance bias and detection bias: Except for one study (34) that indicated the use of single blinding, the other studies did not indicate whether the blind method was used. However, considering that these studies all used objective outcome indicators and the results were not interfered by the researchers and participants. Therefore, these studies were identified as “low risk.” (4) Attrition bias: all included studies had no missing data, so the risk of attrition bias was considered “low.” (5) Reporting bias and other bias: None of the studies had enough information to assess whether there were selective reports and other risks of bias, so they were identified as “unclear risks.” All risk bias assessment data were shown in **Figure 2**.

## Outcomes Measures

### Clinical Total Effective Rate

**Figure 3:**
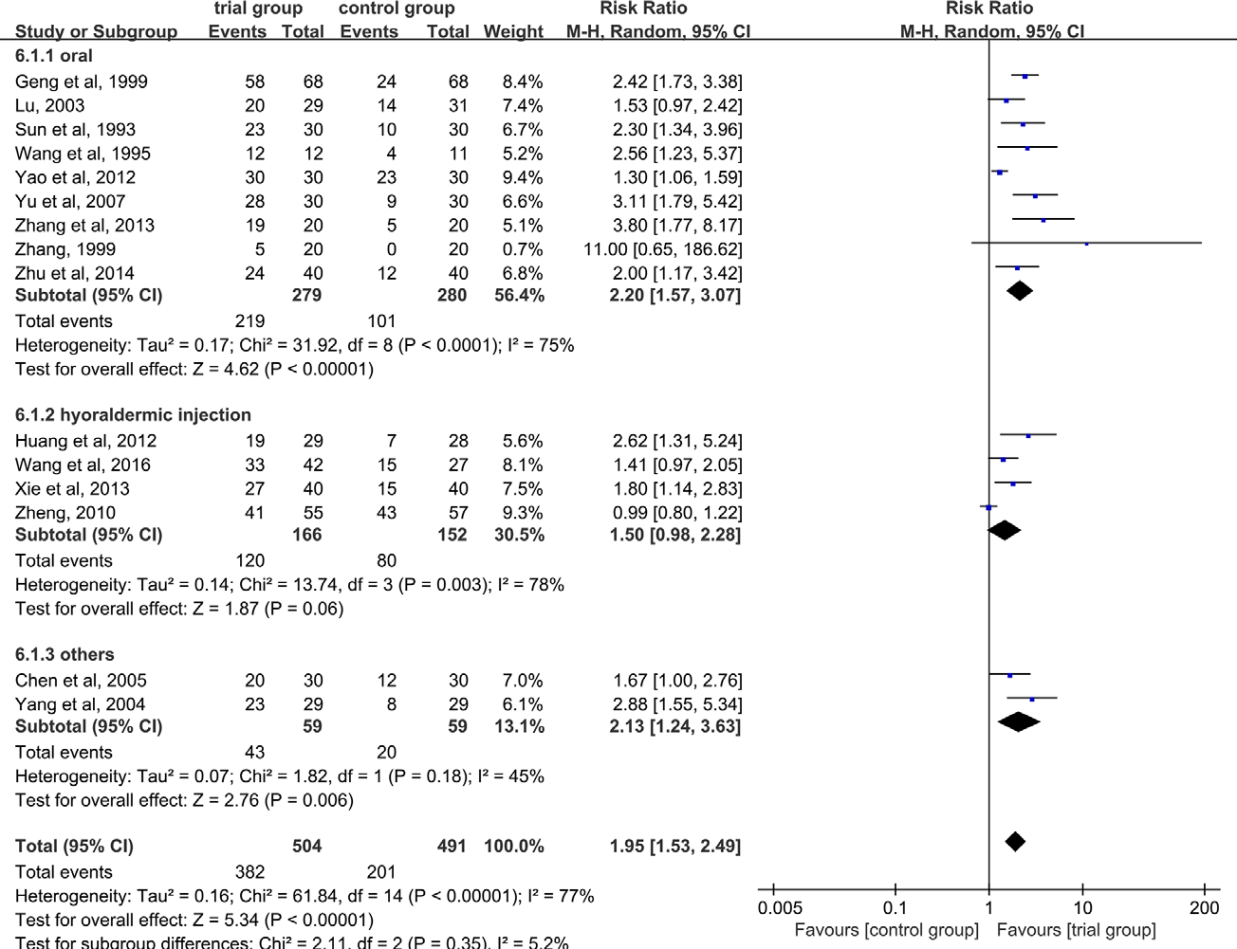
Subgroup analysis of the clinical total effective rate according to the different drug treatment methods in the control group.

Fifteen studies reported effective rate (24–28, 30, 32–40). Due to the heterogeneity of the data (I^2^ = 77%, P <0.00001), random-effect model was adopted. The results showed that the clinical effective rate of ST36 acupoint injection with dexamethasone was significantly higher than that of the CWM treatment group (504/491) (RR=1.95, 95% CI [1.53, 2.49], P <0.00001, **Figure 3**).

**Figure 4:**
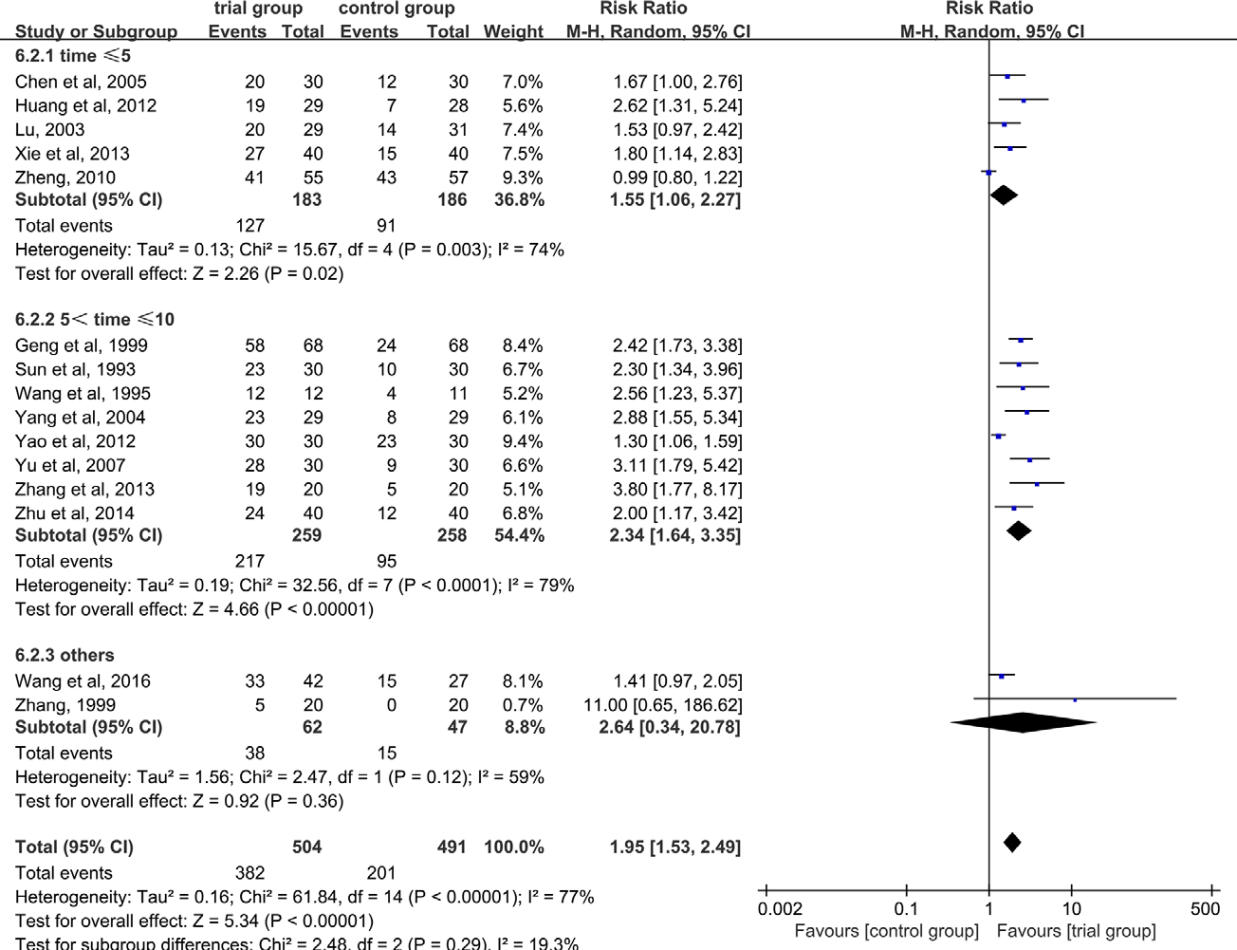
Subgroup analysis of the clinical total effective rate according to different treatment time.

**Figure 5:**
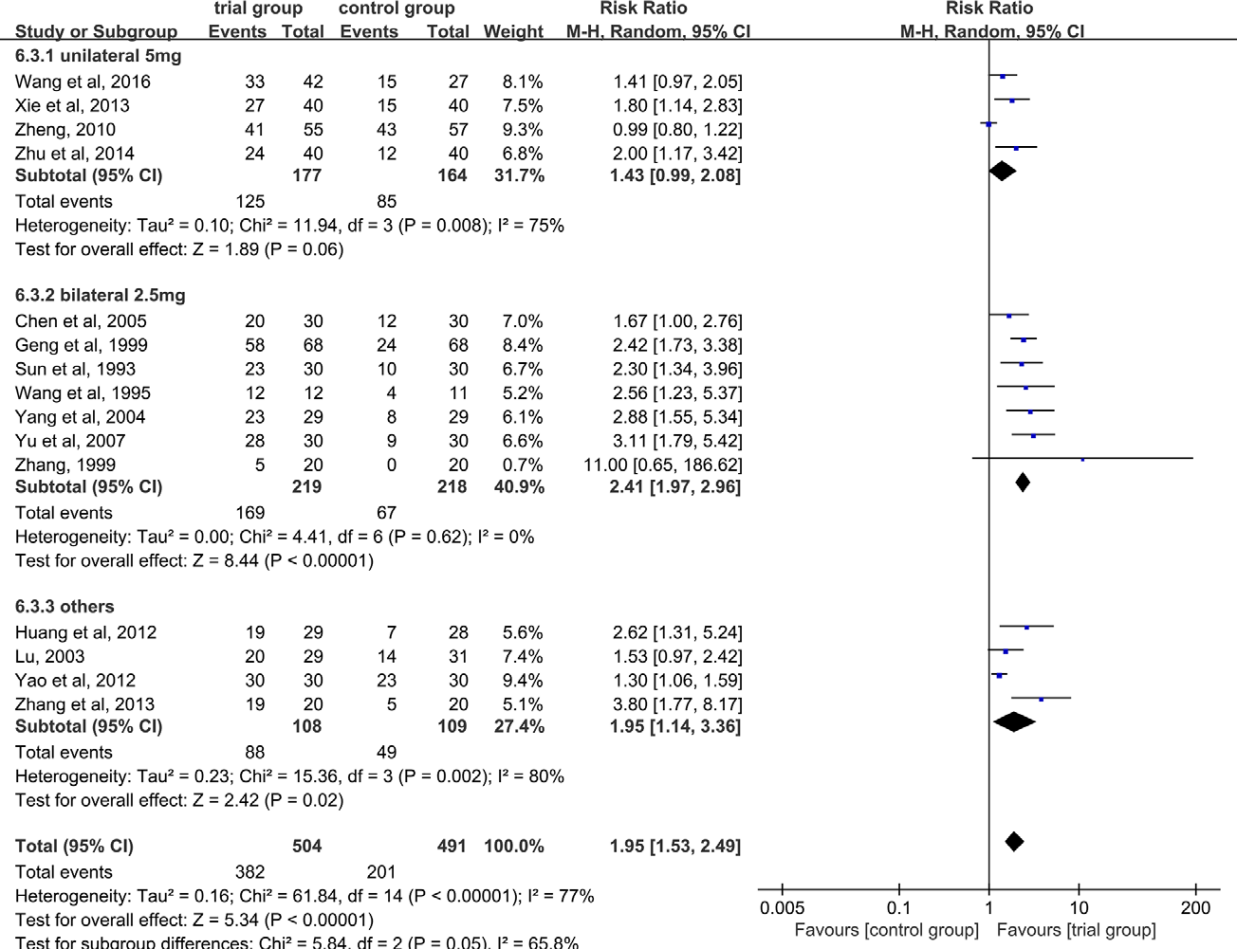
Subgroup analysis of the clinical total effective rate according to the number of acupoints injected with dexamethasone.

Due to the heterogeneity among studies, we conducted a subgroup analysis of the clinical total effective rate according to the different drug treatment methods in the control group (oral or hypodermic injection or others) (**Figure 3**). The results showed that when the control group was treated with oral or hypodermic injection, the heterogeneity among the studies was high (I^2^ = 75%; I^2^ = 78%), When other treatment methods were used, the heterogeneity among the studies was relatively low (I^2^ = 45%). When the control group adopts oral or other treatment methods, the clinical total effective rate of the trial group is higher than that of the control group [RR=2.20, 95% CI [1.57, 3.07], P <0.00001; RR=2.13, 95% CI (1.24, 3.63), P=0.006], when the control group was treated with hypodermic injection, the results was not statistically significant (RR=1.50, 95% CI [0.98, 2.28], P=0.06). We also conducted a subgroup analysis of the clinical total effective rate according to different treatment time (time ≤ 5 or 5<time ≤ 10 or others) (**Figure 4**). The results showed that the heterogeneity of the three subgroups were high (I^2^ = 74%; I^2^ = 79%; I^2^ = 59%). When we analyzed the effective rate of treatment time ≤ 5 days and 5< treatment time ≤10 days, the clinical total effective rate of the trial group was higher than that of the control group [RR=1.55, 95% CI (1.06, 2.27), P=0.02; RR= 2.34, 95% CI (1.64, 3.35), P <0.00001], When we analyzed the effective rate of other treatment times, the result was not statistically significant [RR=2.64, 95% CI (0.34, 20.78), P=0.36]. In addition, we also conducted a subgroup analysis of the clinical total effective rate according to the number of acupoints injected with dexamethasone (unilateral 5 mg or bilateral 2.5 mg or others) (**Figure 5**). The results showed that in the subgroup of unilateral ST36 acupoint injection with 5 mg dexamethasone and others, the heterogeneity among studies were high (I^2^ = 75%; I^2^ = 80%), the heterogeneity among studies was low in the subgroup of bilateral ST36 acupoint injection with 2.5 mg dexamethasone (I^2^ = 0%). In the subgroup of unilateral ST36 acupoint injection with 5 mg dexamethasone, the result was not statistically significant [RR=1.43, 95% CI (0.99, 2.08), P=0.06]. In the subgroup of bilateral ST36 acupoint injection with 2.5 mg dexamethasone and others, the clinical total effective rate of the trial group was higher than that of the control group [RR=2.41, 95% CI (1.97, 2.96), P<0.00001; RR=1.95, 95% CI (1.53, 2.49), P<0.00001].

### WBC Levels

**Figure 6:**
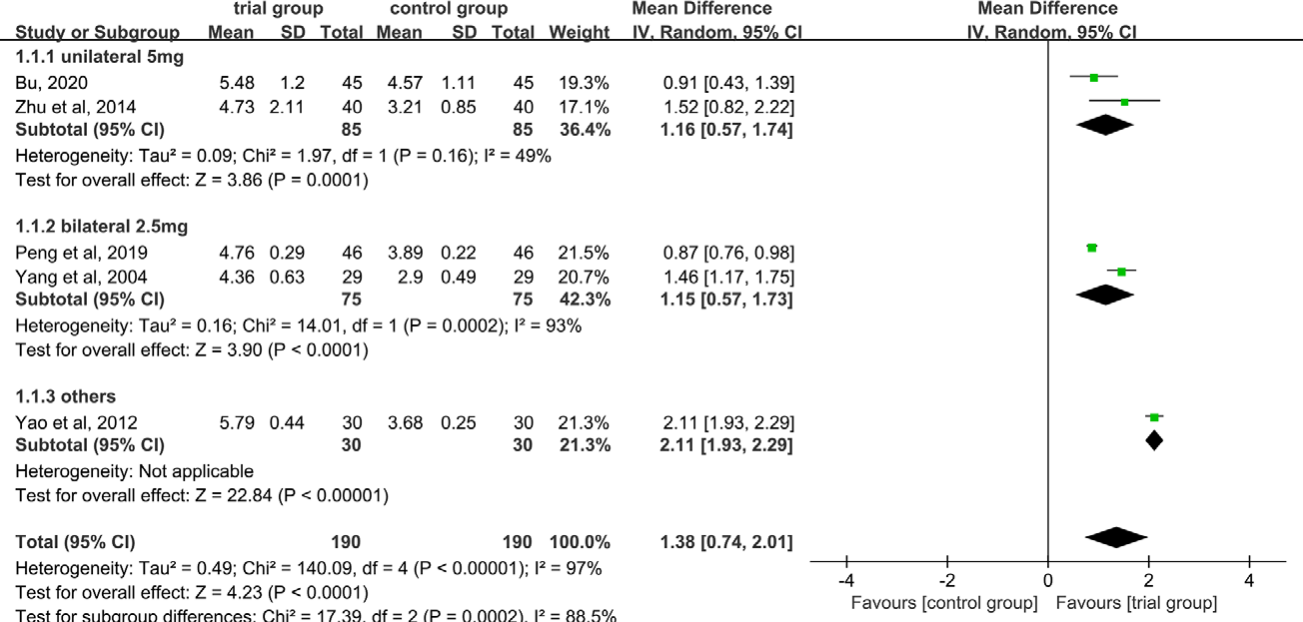
Subgroup analysis of WBC levels according to the number of acupoints injected with dexamethasone.

Five studies provided WBC data (26, 29, 31, 35, 40). Due to the heterogeneity of the data (I^2^ = 97%, P<0.00001), a random-effect model was used. The results showed that the increase in serum WBC levels was greater in the trial group than in the control group (190/190) [MD=1.38, 95% CI (0.74, 2.01), P<0.0001, **Figure 6**].

**Figure 7:**
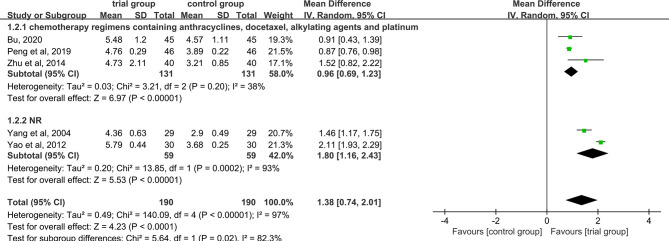
Subgroup analysis of WBC levels according to different chemotherapy regimens.

Due to the high heterogeneity among the studies, we performed a subgroup analysis of WBC levels according to the number of acupoints injected with dexamethasone (unilateral 5 mg or bilateral 2.5 mg or others) (**Figure 6**). The results showed that the heterogeneity among studies was decreased (I^2^ = 49%) in the subgroup of unilateral ST36 acupoint injection with 5 mg dexamethasone, the heterogeneity between studies was still high (I^2^ = 93%) in the subgroup of bilateral ST36 acupoint injection with 2.5 mg dexamethasone. The increase in serum WBC levels was greater in the trial group than in the control group [MD=1.16, 95% CI (0.57, 1.74), P=0.0001; MD=1.15, 95% CI (0.57, 1.73), P<0.0001; MD=2.11, 95% CI (1.93, 2.29), P <0.00001] in the three subgroups. In addition, we also performed a subgroup analysis of WBC levels according to different chemotherapy regimens (**Figure 7**). Studies have shown that chemotherapeutic drugs such as anthracyclines, docetaxel, alkylating agents, and platinum can easily cause blood system toxicity (41). The results showed that in the subgroup of chemotherapy regimens containing anthracyclines, docetaxel, alkylating agents, and platinum, the heterogeneity among studies was low (I^2^ = 38%), in the subgroup where chemotherapy regimens were not reported, the heterogeneity between studies was relatively high (I^2^ = 93%). And the increase in serum WBC levels was greater in the trial group than in the control group [MD=0.96, 95% CI (0.69, 1.23), P<0.00001; MD=1.80, 95% CI (1.16, 2.43), P<0.00001] in the two subgroups.

### Hb Levels

**Figure 8:**
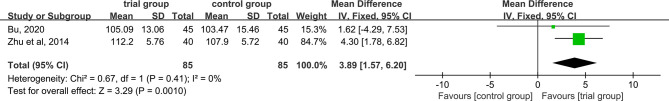
Forest plots of Hb levels.

Two studies provided Hb data (29, 40). As data heterogeneity was not found (I^2^ = 0%, P = 0.41), the fixed-effect model was applied. The results showed that the increase in serum Hb levels was greater in the ST36 acupoint injection with dexamethasone treatment group than in the CWM treatment group (85/85) [MD=3.89, 95% CI (1.57, 6.20), P=0.001, **Figure 8**].

### PLT Levels

**Figure 9:**
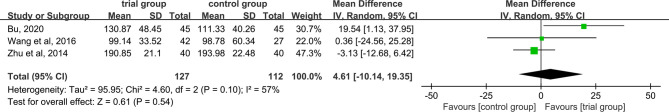
Forest plots of PLT levels.

PLT data were provided in three studies (29, 37, 40). Due to the heterogeneity among the studies (I^2^ = 57%, P=0.1), a random-effect model was used. The results showed that there was no significant difference in serum PLT levels among the trial group and the control group (127/112) [MD=4.61, 95% CI (-10.14, 19.35), P=0.54, **Figure 9**].

### Recovery Time

**Figure 10:**
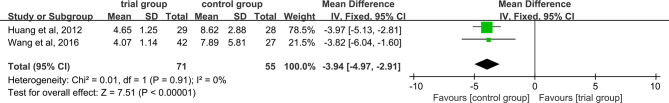
Forest plots of recovery time.

Two studies (34, 37) discussed this result, There was no heterogeneity between studies (I^2^ = 0%, P =0.91), and a fixed-effect model was used. The results showed that ST36 acupoint injection with dexamethasone treatment group significantly reduced the recovery time (71/55) [MD=-3.94, 95% CI (-4.97, -2.91), P<0.00001, **Figure 10**].

### Karnofsky Performance Status

**Figure 11:**
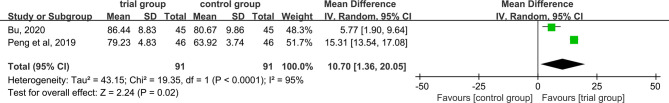
Forest plots of Karnofsky performance status.

KPS can be extracted explicitly from two studies (29, 31). Because of the heterogeneity between the studies (I^2^ = 95%, P <0.0001), a random-effect model was used. The results showed that the KPS score of the ST36 acupoint injection with dexamethasone group was significantly higher than that in the CWM treatment group (91/91) [MD=10.7, 95% CI (1.36, 20.05), P=0.02<0.05, **Figure 11**].

### Adverse Events

Among the included 17 studies, only one study (28) reported adverse events. One patient in the ST36 acupoint injection with dexamethasone group felt unbearable local swelling and pain, while the other patients had no obvious adverse reactions. In the control group, two patients had general aches and discomfort, one patient had bone pain, and one patient had low-grade fever, all of which did not affect the treatment, and were alleviated after the drug was stopped.

### Publication Bias and Sensitivity Analysis

**Figure 12:**
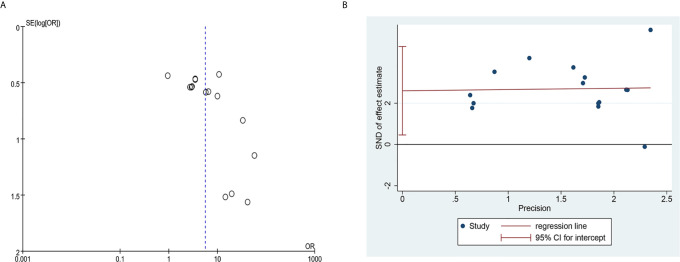
Publication bias plots. **(A)** Funnel plot of clinical total effective rate; **(B)** Egger’s plot of clinical total effective rate.

The funnel plot of the clinical total effective rate (**Figure 12A**) showed an asymmetrical distribution visually, indicating that there might have been publication bias. In addition, the Egger’s test results (**Figure 12B**) were consistent with the funnel plot (t = 4.83, P = 0.0001), further indicating that the effective rate has a publication bias. The research publication bias might be due to the small sample size of the included RCTs, and the lack of negative results might also be the cause of the bias. Since the number of RCTs included in the five studies of WBC count, Hb count, PLT count, recovery time, and quality of life score were less than 10, the publication bias test based on funnel plot and Egger’s test were not performed. We conducted sensitivity analysis on clinical total effective rate, WBC levels, HB levels, PLT levels, recovery time, and KPS. After we excluded each study one by one, there was no significant change in the results, indicating that the result data had good stability.

### Grade Evaluation

GRADE profiler 3.6 software was used to grade the evidence quality of the included studies, and the results showed that three studies (26, 37, 40) used random number table for random allocation, and one study (39) made distribution according to household income and payment method. Other studies claimed to use randomization, but no details on how to randomize were reported. None of the studies clearly stated whether allocation concealment was carried out. Except for one study (34) that indicated the use of single blinding, the other studies did not indicate whether the blind method was used, resulting in some limitations. In addition, except for the large sample size of clinical total effective rate, the sample size of other studies was generally small, and the confidence interval of the study results was relatively wide, which reduced the accuracy of the study. What is more, there might be publication bias in each study result. Therefore, the evidence quality evaluation of clinical total effective rate was low, and the rest of the study results were very low, as shown in **Table 2**.

**Table 2:** Summary of meta-analysis results and GRADE evidence profile.

Quality assessment						No. of patients		RR/MD (95% CI)	P-value	Quality
No.of studies	Risk of bias	Inconsistency	Indirectness	Imprecision	Other considerations	Trial group	Control group			
Clinical total effective rate (better indicated by higher values)										
15	Serious^a^ ^,^ ^b^ ^,^ ^c^	No serious inconsistency	No serious indirectness	No serious imprecision	Reporting bias^e^	382/504 (75.8%)	201/491 (40.9%)	RR 1.95 (1.53 to 2.49)	<0.00001	^⊕⊕^OO LOW
WBC levels (better indicated by higher values)										
5	Serious^a^ ^,^ ^b^ ^,^ ^c^	No serious inconsistency	No serious indirectness	Serious^d^	Reporting bias^e^	190	190	MD 1.38 (0.74 to 2.01)	<0.0001	^⊕^OOO VERY LOW
Hb Levels (better indicated by higher values)										
2	Serious^a^ ^,^ ^b^ ^,^ ^c^	No serious inconsistency	No serious indirectness	Serious^d^	Reporting bias^e^	85	85	MD 3.89 (1.57 to 6.20)	0.001	^©^OOO VERY LOW
PLT Levels (better indicated by higher values)										
3	Serious^a^ ^,^ ^b^ ^,^ ^c^	No serious inconsistency	No serious indirectness	Serious^d^	Reporting bias^e^	127	112	MD 4.61 (−10.14 to 19.35)	0.54	^⊕^OOO VERY LOW
Recovery time (better indicated by lower values)										
2	Serious^a^ ^,^ ^b^ ^,^ ^c^	No serious inconsistency	No serious indirectness	Serious^d^	Reporting bias^e^	71	55	MD −3.94 (−4.97 to −2.91)	<0.00001	^⊕^OOO VERY LOW
KPS (better indicated by higher values)										
2	Serious^a^ ^,^ ^b^ ^,^ ^c^	No serious inconsistency	No serious indirectness	Serious^d^	Reporting bias^e^	91	91	MD 10.70 (1.36 to 20.05)	0.02	^⊕^OOO VERY LOW

WBC, white blood cell; Hb, hemoglobin; PLT, platelet; KPS, Karnofsky performance status; MD, mean difference; RR, risk ratio; CI, confidence interval.

a No detailed of random protocol were reported.

b Lack of allocation concealment.

c No indicate whether the blind method was used.

d Total number of events is less than 400.

e Evaluation of the data revealed publication bias, and it is possible that some “negative” trials were not included in the study.

## Discussion

This study systematically sorted out and analyzed the clinical evidence of ST36 acupoint injection with dexamethasone in the treatment of CIM to better guide clinical practice. Through the analysis of 17 randomized controlled trials, it was suggested that ST36 acupoint injection with dexamethasone had a significant effect on the treatment of CIM, which can effectively increase WBC and Hb levels, shorten the duration of myelosuppression, and improve the quality of life of patients to a certain extent. Heterogeneity was found in the analysis results of the clinical total effective rate, so we conducted a subgroup analysis of the factors that may cause heterogeneity, including the drug treatment method in the control group, treatment time, and the number of dexamethasone injection points. The results of subgroup analysis showed that when the treatment method of the control group was oral or subcutaneous injection or other methods, the heterogeneity still existed. We analyzed this might be related to the different types of specific drugs in the treatment method of the control group. In addition, according to the subgroup analysis of treatment time, heterogeneity still existed, which indicated that treatment time was not a key factor affecting heterogeneity to a certain extent. According to the subgroup analysis based on the number of dexamethasone injection points, the heterogeneity of the subgroup of unilateral ST36 acupoint injection with 5 mg dexamethasone did not decrease significantly, and the result was not statistically significant. However, the heterogeneity of the subgroup of bilateral ST36 acupoint injection with 2.5 mg dexamethasone was significantly reduced, and the result was statistically significant. The possible reason for our analysis is that the number of included studies in the subgroup of unilateral ST36 acupoint injection with 5 mg dexamethasone were small, and the heterogeneity between studies was high, which may affect the study results to a certain extent. We also found heterogeneity in the analysis results of WBC levels, so we conducted a subgroup analysis based on the number of dexamethasone injection points and chemotherapy regimens. The results showed that the heterogeneity was significantly decreased in the unilateral ST36 acupoint injection with 5 mg dexamethasone subgroup, while the heterogeneity was not significantly decreased in the bilateral ST36 acupoint injection with 2.5 mg dexamethasone subgroup. The possible reason for our analysis was that the control group with unilateral ST36 acupoint injection with 5 mg dexamethasone were all oral drugs, while the control group with bilateral ST36 acupoint injection with 2.5 mg dexamethasone were ST36 acupoint injection with normal saline and intramuscular injection with dexamethasone respectively. What is more, the subgroup analysis based on different chemotherapy regimens showed significantly reduced heterogeneity in the subgroup containing anthracyclines, docetaxel, alkylating agents, and platinum, which may be due to the fact that the tumor types of the study subjects in this subgroup were the same and the chemotherapy regimen was relatively uniform. Significant heterogeneity was found in the analysis results of PLT levels and KPS, which may be related to the less included literatures. In this study, except for one study (40) that reported the source of funding, other studies did not clearly report the source of funding. Therefore, it was impossible to determine whether there was a conflict of interest. These potential conflicts of interest may be the cause of publication bias.

ST36 acupoint injection with dexamethasone is essentially a compound treatment method that integrates meridians, acupoints, and drugs. Its treatment of CIM may be the amplification effect produced by the superimposition of the following effects (1): According to the theory of Chinese medicine, Zusanli is the main point for strengthening, and reasonable and moderate acupuncture has the effect of strengthening the body, and reasonable and moderate acupuncture can strengthen the body (42) (2). Studies have proved that the information of acupuncture can be transmitted to the relevant areas of the cerebral cortex through afferent nerves, and then the cortex can be excited to reach the nerve endings of the viscera and related organs along the efferent nerves, stimulating mast cells to release bioactive substances, so as to improve the excitability of the nerve endings and produce sensitive effects (43). For the bone marrow system, the sensitive effect produced by acupuncture can improve the hematopoietic function of the bone marrow and promote the significant increase of blood WBC, RBC, and Hb (44, 45) (3). Glucocorticoids, such as dexamethasone, have a chemotactic effect on various components of blood, which can promote the migration of marginal pool cells to the central pool and redistribute various components of the blood. It can promote neutrophils attached to the edge of small vessels to enter the blood circulation and increase the number of neutrophils. At the same time, dexamethasone can also enhance the body’s emergency response ability and reduce the permeability of capillaries, reduce or alleviate the toxic and side effects of chemotherapy, and improve the symptoms of patients (46).

This study has certain limitations. First, all the included studies were conducted in China, which may lead to potential regional bias. It is difficult to verify whether the efficacy of ST36 acupoint injection with dexamethasone on CIM was applicable to different populations around the world. Second, the methodological quality of the included studies was generally poor, because they reported limited information on allocation sequence generation, allocation concealment, and blinding. Third, there was a potential publication bias in terms of clinical effective rate, which may be due to flaws in the study design of small studies or the failure of the small studies to publish negative results. Fourth, there may be heterogeneity among most of the studies included in the analysis, which are as follows (1): the characteristics of participants were different, such as age, gender, tumor type, physical status, and so on (2); different chemotherapy regimen, frequency, and dose (3); differences in the injection of dexamethasone at Zusanli acupoints, such as drug dosage, frequency, number of acupoints, sensation of deqi, and so on (4); differences in intervention measures of the control group in the included studies, such as the type of drugs, the way of use, and so on. Fifth, most of the included studies did not report adverse events. It is difficult to verify the safety of ST36 acupoint injection with dexamethasone in the treatment CIM. Despite these limitations, this study is the first to systematically evaluate the efficacy of injection of ST36 acupoint injection with dexamethasone on CIM, which may be helpful to clinicians.

## Conclusion

The results of this meta-analysis showed that ST36 acupoint injection with dexamethasone could not only improve the efficacy of the treatment of CIM, but also improve the quality of life of patients. However, because of the risk of bias and low quality of the included trials, further standard, double-blind, multi-center randomized controlled studies are needed to verify the efficacy of ST36 acupoint injection with dexamethasone in the treatment of CIM.

## Data Availability Statement

The original contributions presented in the study are included in the article/**Supplementary Material**. Further inquiries can be directed to the corresponding author.

## Author Contributions

JC and ZL initiated this study and participated in its design. ZL, JC, and JD performed study selection, data extraction, and data analysis. The manuscript was drafted by JC and revised by JC, ZL, and JD. All authors contributed to the article and approved the submitted version.

## Supplementary Material

The Supplementary Material for this article can be found online at: https://www.frontiersin.org/articles/10.3389/fonc.2021.684129/full#supplementary-material

## Conflict of Interest

The authors declare that the research was conducted in the absence of any commercial or financial relationships that could be construed as a potential conflict of interest.

## Abbreviations

CIM, chemotherapy-induced myelosuppression; CWM, conventional western medicine; WBC, white blood cell; Hb, hemoglobin; PLT, platelet; rhG-CSF, recombinant human granulocyte colony stimulating factor; rHuTPO, recombinant human thrombopoietin; rhEpo, recombinant human erythropoietin; RBC, red blood cell; RCTs, randomized controlled trials; CNKI, China National Knowledge Infrastructure; VIP, Chinese Scientific Journal Database; rhIL-11, recombinant human interleukin-11; KPS, Karnofsky performance status; MD, mean difference; RR, risk ratio; CI, confidence interval; I^2^, I-square.
