# Supplementary material for: Zusanli (ST36) Acupoint Injection With Dexamethasone for Chemotherapy-Induced Myelosuppression: A Systematic Review and Meta-Analysis
Source: Front Oncol. 2021 Jul 6;11:684129. doi: 10.3389/fonc.2021.684129 (PMC8291031; doi:10.3389/fonc.2021.684129)
Supplement: Supplementary file 1 [file DataSheet_1.docx]

**Supplement 1 shows the search strategy used for the systematic review**

Search of Cochrane Library

Date Run: 31/12/2020 09:43:49

Comment:

ID Search Hits

#1 (zusanli) OR (ST36) 1214

#2 (acupoint injection) 519

#3 (dexamethasone) 12206

#4 (chemotherapy) 81899

#5 (myelosuppression) OR (leukocytopenia) OR (thrombocytopenia) OR (decreased hemoglobin) OR (anemia) 32989

#6 #1 AND #2 AND #3 AND #4 AND #5 0

Search strategy for Wangfang

(abstract: "myelosuppression" or " leukocytopenia " or " decreased hemoglobin " or " anemia " or " thrombocytopenia ") and (theme: " zusanli " and " acupoint injection " and " chemotherapy ") . 116 literatures were searched.

Search strategy for China National Knowledge Infrastructure (CNKI)

(AB = myelosuppression OR AB = leukocytopenia OR AB = decreased hemoglobin OR AB = anemia OR AB = thrombocytopenia) AND (SU = zusanli AND SU = acupoint injection AND SU = chemotherapy) .66 literatures were searched.

Search strategy for Wangfang

(摘要: "骨髓抑制" or "白细胞减少" or "血红蛋白减少" or "贫血" or "血小板减少") and (主题: "足三里" and "穴位注射" and "化疗") . 116 literatures were searched.

知网：(AB =骨髓抑制 OR AB =白细胞减少 OR AB = 血红蛋白减少 OR AB =贫血 OR AB =血小板减少) AND (SU =足三里 AND SU =穴位注射 AND SU =化疗) .66 literatures were searched.
